# Automatic Cell Type Annotation Using Marker Genes for Single-Cell RNA Sequencing Data

**DOI:** 10.3390/biom12101539

**Published:** 2022-10-21

**Authors:** Yu Chen, Shuqin Zhang

**Affiliations:** 1 School of Mathematical Sciences, Fudan University, Shanghai 200433, China; 2 Key Laboratory of Mathematics for Nonlinear Science (Ministry of Education), Fudan University, Shanghai 200433, China; 3 Shanghai Key Laboratory for Contemporary Applied Mathematics, Fudan University, Shanghai 200433, China

**Keywords:** cell type annotation, marker genes, scRNA-seq

## Abstract

Recent advancement in single-cell RNA sequencing (scRNA-seq) technology is gaining more and more attention. Cell type annotation plays an essential role in scRNA-seq data analysis. Several computational methods have been proposed for automatic annotation. Traditional cell type annotation is to first cluster the cells using unsupervised learning methods based on the gene expression profiles, then to label the clusters using the aggregated cluster-level expression profiles and the marker genes’ information. Such procedure relies heavily on the clustering results. As the purity of clusters cannot be guaranteed, false detection of cluster features may lead to wrong annotations. In this paper, we improve this procedure and propose an Automatic Cell type Annotation Method (ACAM). ACAM delineates a clear framework to conduct automatic cell annotation through representative cluster identification, representative cluster annotation using marker genes, and the remaining cells’ classification. Experiments on seven real datasets show the better performance of ACAM compared to six well-known cell type annotation methods.

## 1. Introduction

The development of single-cell RNA sequencing (scRNA-seq) technology has provided the opportunity for studying genes’ expression at each single-cell level [1]. It has greatly advanced the understanding of biology and medicine in many aspects by analyzing the transcriptome-wide cell-to-cell variations. For example, investigation of the heterogeneity of different cell types in cancer ecosystems contributes to studying the disease progression and response to therapy [2,3,4,5], and exploration of the cell type transitions benefits studying the cell-state progression in the developing embryos [6,7]. With the wide applications of scRNA-seq technology, more and more scRNA-seq data from different platforms are being generated.

Annotation of the cell types plays an essential role in scRNA-seq data analysis. Several computational methods have been proposed for automatic annotation [8,9,10,11,12,13,14,15,16,17,18,19,20]. According to the databases used for conducting annotation, such methods can be divided into two categories. One is to take the previously annotated scRNA-seq database as reference for labelling the unannotated cells (reference scRNA-seq-data-based) [8,9,11,13,14,18,21]. Additionally, the other category is to directly use the marker genes to annotate the cells (marker-gene-based) [16,17,19,22].

The reference scRNA-seq-data-based cell type annotation methods can be divided into several modelling frameworks. Some of these methods map the unannotated cells to the previously annotated reference datasets using selected features, and then assign them the cell types according to their nearest neighbors based on some similarity measures. Such methods include SingleR [9], scmap [14], scMatch [11], cellHarmony [23], SeuratTransfer [21], and so on. Some other methods belonging to this category directly train a supervised learning model in the annotated reference database, and then predict the cell types of those unannotated, for example, scPred [8], CHETAH [13], and scDeepsort [18]. Deep learning methods have also been proposed on the basis of the annotated scRNA-seq database, such as MARS [10], and ItClust [12]. Since heterogeneity exists between different datasets, this category of methods puts forward high requirements for the cell type matching across different datasets. Some methods are developed using the annotated cells from the same dataset to infer the cell types of the remaining, for example, CASSL [24]. For such methods, to obtain the labels of part of the cells is still the cell type annotation problem.

The marker-gene-based cell type annotation methods also fall into different types. CellAssign takes into account the prior knowledge of cell type specific marker genes into the proposed probabilistic model to infer the type of each cell [19], which is unstable due to the large noise in scRNA-seq data. Garnett first labels a number of representative cells by scoring the marker genes, and then uses logistic regression with elastic net to classify the remaining cells [16]. CALLR improves Garnett by proposing a semi-supervised model for classifying the cells [22]. The performance of these two methods is greatly dependent on the representative cells selected using TD-IDF, which does not work stably for scRNA-seq data from different platforms. SCSA calculates cell type scores of each cluster by adding up re-scaled log2-based fold change values of differentially expressed marker genes. Clusters are then annotated as the cell type with the highest cell type score [25]. scCATCH first obtains the meta information of cell clusters, then by paired comparison of the groups, the potential marker genes for each cluster are identified. The cell types are determined by matching them with the validated marker genes [17]. Similar to scCATCH, deCS [20] annotates the cells using Fisher’s exact test to choose the maximum overlap between the differentially expressed genes found in different clusters and the marker genes, though it can also annotate the cells with the annotated reference scRNA-seq dataset. SCSA, scCATCH, and deCS all assume the clusters are well defined, which may not be the truth in real data analysis. Current clustering methods are still far from sufficient for accurate annotation.

In this work, to overcome the problems existing in the marker-gene-based annotation methods, we propose an Automatic Cell type Annotation Method (ACAM) based on marker genes’ information with no annotated cells needed. This method first finds the representative clusters by searching for the consistent subgroups across the results of several popular clustering methods, such as the method in Seurat [26], SC3 [27], CIDR [28], t-SNE+k-means [29], and SIMLR [30]. Such a technique guarantees that the cells in the same cluster have very high probabilities of being from the same cell type. Then, by selecting the features that discriminate one cluster from all the remaining cells, the potential marker genes are identified. The cell types are determined by defining a cell type importance score to match these potential marker genes with the validated ones. For those cells that do not belong to any of these clusters, we use *k*-nearest neighbors to determine their cell type. We did experiments on seven real-world datasets, and compared the results with six well-known methods. Results show the better performance of ACAM. ACAM fits well with our intuition for cell type annotation, takes advantage of the properties of scRNA-seq data, and is easily implementable.

## 2. Materials and Methods

### 2.1. Datasets

Seven real-world datasets were selected for comparison and testing. Information of the datasets is given in Table 1. All the cells in these datasets have known annotated labels. These datasets were chosen from various platforms. Dataset Chen [31] and Xin [32] were generated using Fluidigm C1 system. Dataset Kidney, Mammary [33], and PBMC [26] were generated using 10× Genomics. Other datasets were chosen from platforms, such as SeqWell and DropSeq [34,35]. The selected datasets have various magnitudes, ranging from 203 cells to 20,679 cells. Several tissues from both human and mouse were selected to demonstrate the overall performance of the methods.

According to the cell type annotation method Garnett [16], consensus cell types were merged together. To be specific, ‘AT1 cells’, ‘AT2 cells’, and ‘alveolar bipotent progenitors’ were merged into ‘alveolars’. ‘Ciliated cells’, ‘clara cells’, and ‘dividing cells’ were merged into ‘ciliated cells’. ‘Stromal cells’ and ‘fibroblasts’ were merged into ‘fibroblasts’. ‘Neutrophils’, ‘eosinophils’, ‘basophils’, and ‘granulocytes’ were merged into ‘granulocytes’. ‘Nuocytes’ and ‘T cells’ were merged into ‘T cells’. ‘Dentritic cells’, ‘monocyte progenitor cells’, ‘monocytes’, and ‘macrophages’ were merged into ‘monocytes’. Consensus cell types ‘Cajal-Retzius cells’ and ‘GABAergic cells’ were merged into ‘neurons’ in dataset Wu [36].

In our study, we use the marker gene database CellMatch [17], which is derived from several popular database, such as CellMarker [37], MCA [38], CancerSEA [39], and the CD Marker Handbook [40]. The corresponding species and tissue of the dataset are selected in the subjects ‘speciesType’ and ‘tissueType’, and the ‘Single-cell sequencing’ entry is chosen in ‘markerResource’. Then, cell types and their markers are chosen from the subjects ‘cellMarker’ and ‘shortname’, respectively. Markers for each cell type are then collected as input of the proposed method.

### 2.2. Methods

**Figure 1:**
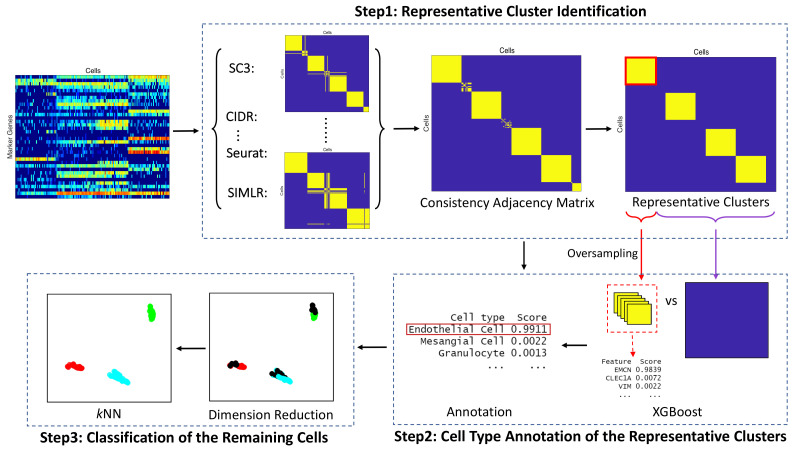
The workflow of ACAM. The input is a log-normalized expression matrix for marker genes only. Markers are selected from the database CellMatch. Step 1. Apply different clustering methods, such as SC3 [27], CIDR [28], t-SNE+k-means [29], and SIMLR [30] to conduct clustering independently, and define the consistency adjacency matrix. Louvain algorithm is applied to identify the representative clusters. Step 2. Apply XGBoost to each representative cluster versus all the remaining cells to obtain each feature’s importance score. Clusters are annotated by the maximum cell type score, which is defined as the sum of the importance score for all the features in each cell type. Step 3. Classify the remaining cells using *k*-nearest neighbors (*k*NN) after dimension reduction.

In this subsection, we present the proposed automatic cell type annotation method ACAM. The workflow of ACAM is shown in Figure 1.

Let ${\tilde{X}}_{p\times n}$ be the scRNA-seq gene expression matrix with *p* genes and *n* cells, which is firstly log-normalized after size factor adjustment for read depth [16]. We denote U as the cell set with |U|=n. Let $G=\{G_{1},G_{2},\ldots ,G_{T}\}$ be the list of marker genes for the considered species and tissue retrieved from the known database, where $G_{t}$ denotes the list of markers for cell type *t*. We keep the related marker genes’ expression in $\tilde{X}$ only, and remove those with zero expression across all the cells. Cells with zero expression across all marker genes are annotated as ‘unknown’, and are removed directly. Without confusion, we still use U and *n* to denote the remaining cell set and the remaining number of cells. The resulted data matrix is denoted as *X*, which is of size M×n, where *M* is the number of marker genes for all considered cell types.

#### 2.2.1. Representative Cluster Identification

Annotation accuracy usually heavily depends on clustering results. Each existing clustering method is insufficient for accurate annotation. Thus, to guarantee that cells from the same cluster are of the same cell type with high probability, we implement several state-of-the-art clustering methods independently, and the consensus subgroups are identified as the representative clusters. We note that any clustering method can be chosen here.

In this work, we choose five clustering methods, which include SC3 [27], CIDR [28], Seurat [41], t-SNE [29] +k-means, and SIMLR [30] according to [42]. After applying these methods, we obtain five different partitions of the cells: $C_{i}=\left\{C_{i1},\ldots ,C_{ik_{i}}\right\}$, i=1,2,…,5 corresponding to the five clustering methods. $C_{il}$ denotes the *l*-th cluster for the *i*-th clustering method, and $k_{i}$ is the corresponding number of clusters. A brief description of five clustering methods is put in Appendix A. We then choose four of the five clustering results having the largest difference according to the variations in the pairwise Adjusted Rand Index (ARI) between any two different clustering methods [43]. To be specific, a 5×5 ARI matrix *R* is constructed by calculating

$$R\left(i,j\right)=\mathrm{ARI}\left(C_{i},C_{j}\right).$$

For each row of *R*, we calculate the variance, and remove the clustering of the minimum variance. Without confusion, we use 1, 2, 3, and 4 to denote the four remaining methods.

To figure out the consistent clusters of the four methods, we construct graphs corresponding to the clustering results, and apply community detection methods. Let $A_{i}$ (i=1,2,3,4) be the adjacency matrix of the graph corresponding to the results of clustering method *i*, where

$$A_{i}\left(u,v\right)=\left\{\begin{array}{cc} 1, & \mathrm{cell}\;\;u,v\;\;\mathrm{from}\;\;\mathrm{the}\;\;\mathrm{same}\;\;\mathrm{cluster},\\ 0, & \mathrm{otherwise}. \end{array}\right.$$

The information from the four clustering methods is combined by adding $A_{i}$’s up and the consistency adjacency matrix $A^{con}$ is defined as follows:

$$\tilde{A}=\sum_{i=1}^{4}A_{i},\;A^{con}\left(u,v\right)=\{\begin{array}{cc} 1, & \tilde{A}\left(u,v\right)=4,\\ 0, & \mathrm{otherwise}. \end{array}$$

We apply Louvain algorithm [44] to $A^{con}$ to identify the communities, which are taken as the consistent clusters. Clusters with size larger than a threshold are finally set as the representative clusters, which are denoted as $P=\{P_{1},P_{2},\ldots ,P_{C}\}$, where *C* is the number of representative clusters. In our experiments, we set the threshold to be 10.

#### 2.2.2. Cell Type Annotation of the Representative Clusters

In our setting, no annotated cells are given, thus supervised learning methods cannot be directly applied to label the unannotated cells. We assign each representative cluster a temporary label, and apply supervised learning methods to extract the features that discriminate it from the remaining cells. Then we match the extracted features to the known cell type associated marker genes, and assign the most probable cell type to the cluster.

Since marker genes of one particular cell type are more likely to be highly expressed in the cells of the type, while comparatively merely expressed in other cell types, extreme gradient boosting (XGBoost) [45] is a good choice for extracting the important features. XGBoost is known as a fast, flexible, and efficient gradient boosting tree skilled in tackling highly sparse data, and performs very well in many classification problems. It constructs the tree by splitting the features into two nodes according to each feature’s value. This fits well with the property of marker genes. Here, we apply XGBoost to extract the features that discriminate each representative cluster $P_{c}$ (target group) from all the remaining cells $U-P_{c}$ (adversarial group), namely the whole set with the subset $P_{c}$ removed. According to the property of marker genes, to make sure the features having high feature importance score are the marker genes of $P_{c}$, each gene’s mean expression level is compared between the target group and the adversarial group, and those having lower mean expression values in the target group are removed before putting into the XGBoost model. Since normally the size of $P_{c}$ is much smaller than that of $U-P_{c}$, to balance the size of the two groups, we adopt the oversampling technique to make the target group have a similar size to the adversarial group. Specifically, we randomly select the cells belonging to $P_{c}$, until the size of the target group is the same as that of the adversarial group. Hinge loss is chosen as the objective and the tree depth is set to 1 in the XGBoost model. We implement XGBoost from R package xgboost. Feature importance $w_{m}$ of each marker gene *m* can be obtained after running XGBoost. Then the feature importance for each cell type *t* is calculated by

$$Score_{t}=\sum_{m\in G_{t}}w_{m},$$

The representative cluster is annotated as the cell type $t_{0}$, where $t_{0}$ is the cell type that maximizes $Score_{t}$ for all *t*.

#### 2.2.3. Classification of the Remaining Cells

We apply *k*-nearest neighbors (*k*NN) to annotate the cells that do not belong to any representative cluster. Before doing *k*NN, we apply uniform manifold approximation and projection (UMAP) [46], which efficiently conducts dimension reduction and preserves the high dimensional structure, to project the cells into two-dimensional space for the following classification and visualization. *k*NN is then applied to assign the remaining cells to the annotated representative clusters. We simply set *k* to be 1.

We put the overall procedure in Algorithm 1.
**Algorithm 1** ACAM: Automatic Cell type Annotation Method.**Input:** The pre-processed data matrix *X*, marker gene set G1:Initialize thresh=10, k=12:$C_{1}\leftarrow SC3\left(X\right)$; $C_{2}\leftarrow CIDR\left(X\right)$; $C_{3}\leftarrow Seurat\left(X\right)$;$C_{4}\leftarrow t$-SNE+k-means(X); $C_{5}\leftarrow SIMLR\left(X\right)$3:$R_{i,j}\leftarrow ARI\left(C_{i},C_{j}\right),\;i=1,\ldots ,5$4:Remove the method of $\arg\min_{i}var\left(R_{i,\cdot }\right)$5:$A_{i}$: $A_{i}\left(u,v\right)\leftarrow \left\{\begin{array}{cc} 1, & u,v\;\;\mathrm{from}\;\;\mathrm{the}\;\;\mathrm{same}\;\;\mathrm{cluster},\\ 0, & \mathrm{otherwise}. \end{array}\right.$6:$\tilde{A}\leftarrow \sum_{i=1}^{4}A_{i}$7:$A^{con}$: $A^{con}\left(u,v\right)\leftarrow \{\begin{array}{cc} 1, & \tilde{A}(u,v)=4,\\ 0, & \mathrm{else}. \end{array}$8:$P=\left\{P_{1},P_{2},\ldots ,P_{C}\right\}\leftarrow Louvain\left(A^{con},thresh\right)$9:**for** c = 1,…,C **do**11:   ${\tilde{P}}_{c}\leftarrow Oversample\left(P_{c}\right)$11:   Select *m* with $mean\left(X\left[m,{\tilde{P}}_{c}\right]\right)>mean\left(X\left[m,U-P_{c}\right]\right)$12:   $w_{m}\leftarrow XGBoost\left({\tilde{P}}_{c},U-P_{c}\right),m\in G_{t},\;t=1,\ldots T$13:   $Score_{t}=\sum_{m\in G_{t}}w_{m},\;t=1,\ldots ,T$14:   $y_{u}\leftarrow \mathrm{cell}\;\mathrm{type}\;\;t_{0}$: $t_{0}=\arg\max_{t}\left(Score_{t}\right),\;u\in P_{c}$15:**end for**16:$y_{u}\leftarrow kNN\left(X\left[,P\right],k\right),\;u\in U-P$**Output:** 
Cell labels *y*

### 2.3. Results Evaluation Metrics

We choose four metrics: accuracy, balanced accuracy, macro F1-score, and Matthews correlation coefficient (MCC) to measure the performance. Let the total number of cell types be *T* and the total number of cells be *n*. Let $TP_{t}$, $FP_{t}$, and $FN_{t}$ denote the true positive, false positive, and false negative for the cell type *t* in the confusion matrix constructed for the underlying true labels and the inferred labels. $Row_{t}$ and $Col_{t}$ denote the *t*-th row and column of the confusion matrix.

- Accuracy: It is defined as the percentage of true positives of the annotations:
  $$Accuracy=\frac{\sum_{t=1}^{T}{TP}_{t}}{n}.$$
- Balanced Accuracy: It is defined as the average Recall of each cell type,
  $$Balanced\;\;Accuracy=\frac{\sum_{t=1}^{T}{Recall}_{t}}{T},$$
  where
  $${Recall}_{t}=\frac{{TP}_{t}}{{TP}_{t}+{FN}_{t}}.$$
- Macro F1-Score: It is defined as the harmonic mean of average Precision and average Recall:
  $$Macro\;F1-Score=2\times \frac{Average\;\;Precision\times Average\;\;Recall}{Average\;\;Precision+Average\;\;Recall}$$
  where
  $${Precision}_{t}=\frac{{TP}_{t}}{{TP}_{t}+{FP}_{t}}.$$
- Matthews Correlation Coefficient (MCC): It takes into account the true and false positives and negatives and is generally regarded as a balanced measure which can be used even if the classes are of very different sizes:
  $$MCC=\frac{\sum_{t=1}^{T}{TP}_{t}\times n-\sum_{t=1}^{T}{Col}_{t}\times {Row}_{t}}{\sqrt{\left(n^{2}-\sum_{t=1}^{T}{Col}_{t}^{2}\right)\left(n^{2}-\sum_{t=1}^{T}{Row}_{t}^{2}\right)}}.$$

The detailed definitions of these metrics can be found in [47].

## 3. Results

We evaluated ACAM using seven real-world datasets, and compared with six well-known cell type annotation methods, especially the marker-gene-based methods.

### 3.1. Comparison Methods

ACAM was compared with four marker-gene-based methods: CellAssign [19], deCS [20], Garnett [16], SCSA (SCSA_Scran [48], and SCSA_Seurat [25,26]). To give a more general picture of the annotation methods, we also added two well-known reference scRNA-seq-data-based methods: SeuratTransfer [21] and SingleR [9] into comparisons.

- **CellAssign** It takes into account the prior knowledge of marker genes into a probabilistic model to estimate cell types with parameters selected by the maximum a posteriori probability, and google tensorflow is used in EM step.
- **deCS** It first conducts clustering by Seurat [26]. Differentially expressed genes of clusters are then extracted using function FindAllMarkers in R package Seurat. It then annotates clusters as the cell type with the maximum overlap between cell type markers and the differentially expressed genes.
- **Garnett** It first chooses representative cells by aggregating marker scores from the TF-IDF matrix, and then trains the logistic regression model with elastic net to classify the remaining cells, regarding the representative cells as training set.
- **SCSA** Similar to deCS, SCSA first conducts clustering by Seurat [26]. Differentially expressed genes of clusters are extracted using the function FindAllMarkers in R package Seurat (SCSA_Seurat) and the function findMarkers in R package Scran (SCSA_Scran). SCSA calculates cell type scores of each cluster by adding up re-scaled log2-based fold change values (LFC) of differentially expressed marker genes. Clusters are then annotated by the cell type with the highest cell type score.
- **SeuratTransfer** It uses the function TransferData in the R package Seurat. It is a strategy to ‘anchor’ datasets together. By placing both the annotated reference scRNA-seq dataset and the unannotated dataset in a shared low-dimensional space using canonical correlation analysis (CCA), pairwise correspondences between cells from both datasets are identified as anchors by mutual nearest neighbors (MNN). For each cell in the unannotated dataset, it is scored and annotated depending on the distances to anchors.
- **SingleR** It first calculates the Spearman coefficients on variable genes between each unannotated cell and the annotated ones of each type in the reference scRNA-seq data. The same procedure is iteratively performed using the cell types with top correlations in the previous step. The cell is annotated as the type that is left till the last round.

### 3.2. Methods’ Implementation Details

In the representative clustering identification step of ACAM, we implemented the five clustering methods using their corresponding R package. We accelerated the clustering procedure for the datasets of sample size larger than 4000. In SC3 and SIMLR method, the number of cluster was calculated by the function sc3_estimate_k in R package SC3 and function SIMLR_Estimate_Number_of_Clusters in R package SIMLR, respectively. The threshold of the size of representative clusters was normally set to 10, apart from the dataset Chen, which was set to 5 independently due to its small sample size. This parameter can be set manually according to the prior knowledge.

For the marker-gene-based methods: Garnett, CellAssign, deCS and SCSA, CellMatch was selected as the input marker database. For the annotated reference scRNA-seq-data-based methods: SingleR and SeuratTransfer, we constructed the reference dataset by setting the expression of each marker gene in CellMatch database to be 1 in its cell type, and 0 otherwise to fairly compare the annotation capability. The parameters for all the methods were set to default. In the method SeuratTransfer, we changed k.weight ranging from 10 to the maximum, and the one with the highest accuracy was chosen in our comparisons. Note that all the input expression matrices were in the log-normalized form. For the dataset Wu, due to its time and memory cost in the five clustering procedures and the tensorflow procedure in the method CellAssign, we randomly split it into five subsets with equal size to complete the annotation independently, and then summarized the results.

### 3.3. Results

**Figure 2:**
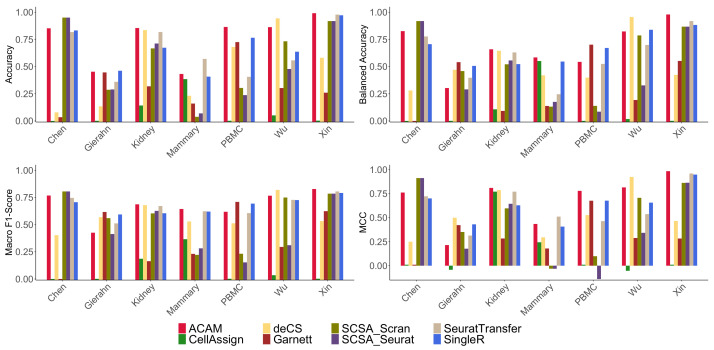
Annotation results comparison. Results of the compared methods using four evaluation metrics: accuracy, balanced accuracy, macro F1-score, and MCC on seven real-world datasets are shown.

Figure 2 shows the cell type annotation results for the compared methods using four measures. ACAM performed stably in all datasets. Most scores of ACAM were ranked first and second. CellAssign did not perform as good as the other methods in our comparison. It failed to find more than 10 true labels in dataset Chen, Xin, Gierahn, and PBMC. Garnett had good performance only in the dataset PBMC and Gierahn, both of which are datasets of human peripheral blood. deCS and SCSA did not have stable annotation scores. deCS reached the best and second best scores in dataset Kidney and Wu, and two SCSA methods reached the best accuracy in dataset Chen. However, deCS obtained the top three worst performance in dataset Chen, Gierahn, and Mammary, which shows the instability of deCS. SingleR also reached high accuracy in all datasets. Though not as good as ACAM, two annotated reference-data-based methods, SeuratTransfer and SingleR reached a stable accuracy in all datasets.

**Figure 3:**
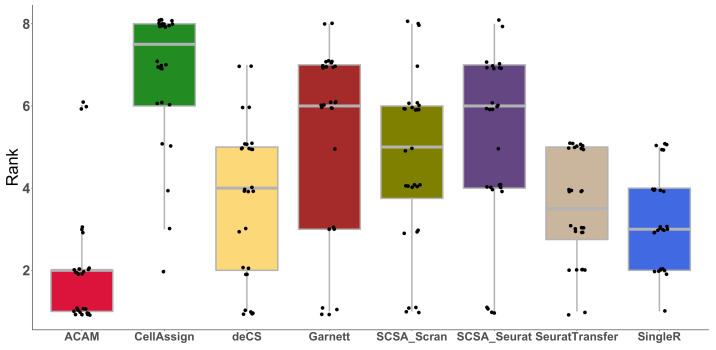
Rank of the compared methods. Boxplot of the rank of each method according to four evaluation metrics: accuracy, balanced accuracy, macro F1-score, and MCC on seven datasets is shown. A lower rank represents better performance (one is the best and eight is the worst).

To give an overall evaluation of all the seven methods, we ranked the methods according to the four metrics in each dataset ranging from one to eight. There are a total of 28 ranks for each method. Lower rank represents better performance (one is the best and eight is the worst). Figure 3 shows the boxplot of all methods. The overall ranks of ACAM are much lower than the other methods, especially the marker-gene-based methods.

**Figure 4:**
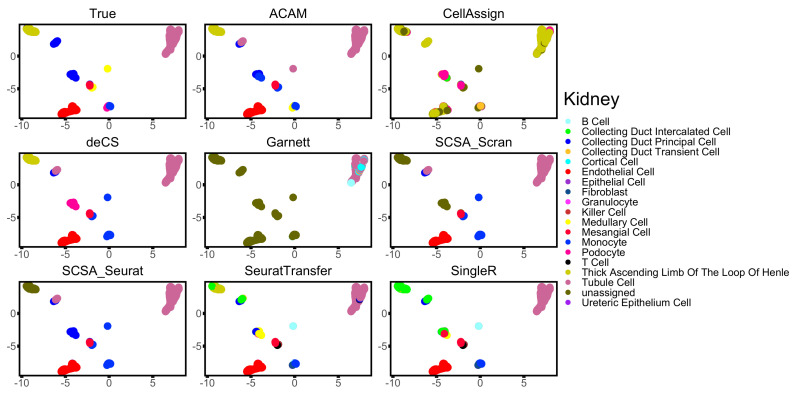
Two-dimensional visualization of the annotation results for dataset Kidney using UMAP.

**Figure 5:**
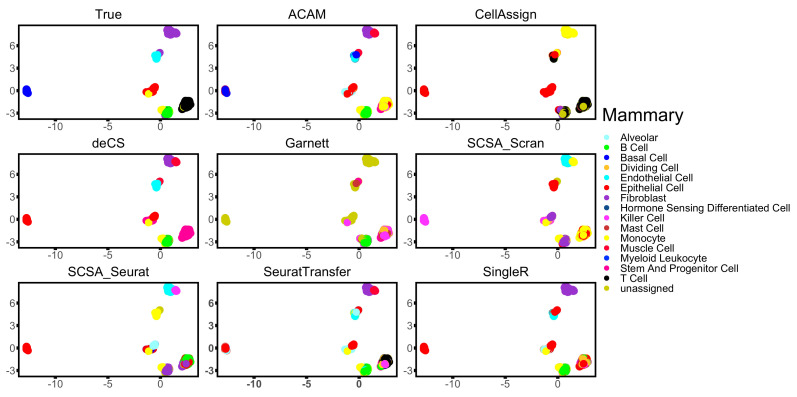
Two-dimensional visualization of the annotation results for dataset Mammary using UMAP.

**Figure 6:**
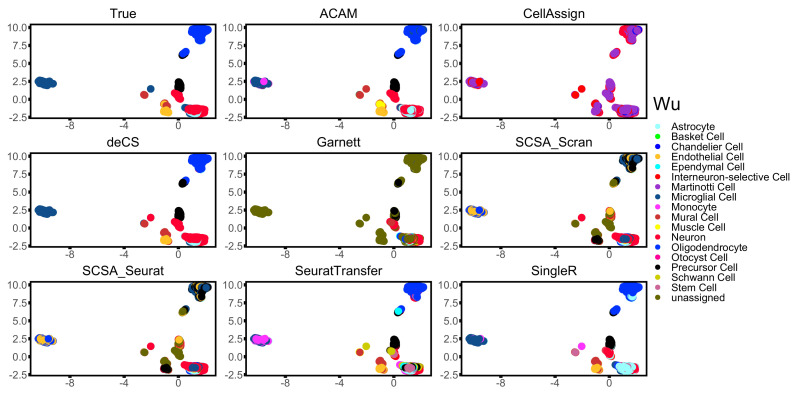
Two-dimensional visualization of the annotation results for dataset Wu using UMAP.

To take a deeper look into the performance of all the compared methods, visualization of three datasets: Kidney, Mammary, and Wu is shown in Figure 4, Figure 5 and Figure 6. ACAM managed to annotate the main clusters stably and correctly, while other marker-gene-based methods did not. CellAssign, which involves a more complicated model and uses iteration technique, failed to tell difference across some cell types of large size. It wrongly annotated most cells into the cell type ‘thick ascending limb of the loop of Henles’ in dataset Kidney (Figure 4) and ‘Martinotti cells’ and ‘neurons’ in dataset Wu (Figure 6). Garnett was able to annotate only a small part of cell types correctly. Most cells were left unassigned in all three datasets (Figure 4, Figure 5 and Figure 6). This should be due to the TD-IDF scoring system used for constructing the training set for the following supervised learning. It may not work well for datasets from various platforms and tissues. deCS and SCSA annotated cells based on the clustering results. deCS wrongly annotated part basel cells of the dataset Mammary (Figure 5). Two SCSA methods failed to correctly annotate lots of cells in dataset Mammary and Wu (Figure 5 and Figure 6). This should be due to the clustering results, which will strongly affect the accuracy of annotations. SeuratTransfer did not perform as stable as SingleR and ACAM. It performed well in dataset Kidney and Mammary, but for dataset Wu, it failed to discriminate the combination of subgroups, and hardly annotated cells in the bottom right corner in Figure 6. In addition, some of the microglial cells was wrongly annotated as monocytes. Though SingleR reached high accuracy in most datasets, it did not perform as well as ACAM. As shown in Figure 4, ACAM correctly labeled ‘thick ascending limb of the loop of Henles’ in dataset Kidney, SingleR, however, failed to annotated them correctly. The same happened for ‘basel cells’ in dataset Mammary and ‘neurons’ in dataset Wu, as shown in Figure 5 and Figure 6, respectively.

## 4. Conclusions and Discussion

In this study, we present an automatic marker-gene-based cell type annotation method: ACAM. It has a clear framework composed of three steps. First, trustworthy representative clusters are identified. Then, a marker-gene-based annotation strategy is designed to perform cell type assignment according to the importance score of the marker genes that discriminate one specific representative cluster from the remaining cells. After all the representative clusters are labeled, *k*NN is applied to annotate the remaining cells outside the representative clusters.

The comparison of ACAM with other methods including both marker-gene-based methods and reference scRNA-seq-data-based methods shows the superiority of ACAM in cell type annotation. ACAM performed better in datasets with different attributes, such as various sample sizes, different species and tissues, and several data generation platforms. The better performance of ACAM against the marker-gene-based methods that conduct annotation based on clustering results indicates that clustering is still an important problem for accurate cell type annotation. In our current setting, though the consistent clusters across several clustering results in ACAM give better annotation, it is at the cost of more computational time. The better performance of ACAM over the marker-gene-based methods that annotate each single cell individually suggests that cluster-level information is more stable, especially when there exists severe noise in the data.

In our current study, each cell is assigned to a known cell type of size greater than a given threshold (10 as default), which may mis-classify the rare cells, and the cells of unknown cell type. How to define the unknown cell types and find the rare cell types according to the marker genes’ expression is still worth further exploration.

## Figures and Tables

**Table 1:** Summary of datasets.

Dataset	Platform	Samples	Cell Types	Species	Tissue
Chen [31]	Fluidigm C1 system	203	3	Mouse	Kidney
Xin [32]	Fluidigm C1 system	1600	4	Human	Pancreatic Islet
Gierahn [34]	Seqwell	3694	5	Human	Peripheral Blood
Wu [35]	DropSeq	20,679	7	Mouse	Brain
PBMC [26]	10×	2638	4	Human	Peripheral Blood
Kidney [33]	10×	2781	8	Mouse	Kidney
Mammary [33]	10×	4481	7	Mouse	Mammary Gland

## Data Availability

ACAM is available as an R package at https://github.com/yuc0824/ACAM (accessed on 30 September 2022). No new data were generated for this study. All data used in this study are publicly available. Datasets below are accessible in GEO with the following accession codes: (a) Chen dataset, GSE99701. (b) Xin dataset, GSE81608. (c) Gierahn dataset, GSM2486333. (d) Wu dataset, GSE103976. (e) Kidney and Mammary dataset, GSE109774. For PBMC dataset, data is available at https://www.10xgenomics.com/ (accessed on 19 October 2021).

## Appendix A

We give a brief description of the five clustering methods used in ACAM.

SC3 [27]: Distance between the cells are calculated using the Euclidean, Pearson, and Spearman metrics to construct distance matrices. Dimensions of three distance matrices are then reduced using either principal component analysis (PCA) or associated graph Laplacian. It does *k*-means clustering on these six metrics. A consensus matrix is then calculated using the Cluster-based Similarity Partitioning Algorithm (CSPA) on these six matrices and it is clustered using hierarchical clustering with complete agglomeration.

CIDR [28]: It imputes dropout candidates, constructs the dissimilarity matrix and maps it into a low-dimensional space by the principal coordinate analysis (PCoA) method. It finally clusters cells by the hierarchical clustering.

Seurat [41]: It identifies clusters of cells by a shared nearest neighbor (SNN) modularity optimization based clustering algorithm. It first calculate *k*-nearest neighbors and construct the SNN graph. It then optimize the modularity function to determine clusters.

t-SNE [29] +k-means: It conducts t-SNE to reduce the dimension and then conducts clustering by *k*-means method.

SIMLR [30]: It conducts the multi-kernel learning framework to obtain a sparse similarity matrix. A spectral clustering algorithm is then applied, which is very effective for clustering sparse similarities and scaling cells.
